# Novel compound heterozygous *TTN* gene variants with additional potential contributory mutations in two sisters with severe scoliosis: A case report

**DOI:** 10.1016/j.gendis.2024.101477

**Published:** 2024-12-03

**Authors:** Huaiyuan Wang, Shengjie Li, Weiyun Chen, Jianxiong Shen

**Affiliations:** a Department of Anesthesiology, Peking Union Medical College Hospital, Beijing 100730, China; b Biomedical Engineering Facility of National Infrastructures for Translational Medicine, Peking Union Medical College Hospital, Beijing 100730, China; c Department of Orthopedic Surgery, Peking Union Medical College Hospital, Beijing 100730, China

Titin, the largest known protein in nature, is a giant sarcomeric protein that plays essential architectural, developmental, and regulatory roles in striated muscles. Mutations in the *TTN* gene (MIM: 188840) that encodes titin are related to a broad range of muscle diseases known as titinopathies, with diverse clinical manifestations including weakness, contractures, scoliosis, respiratory failure, and cardiomyopathy.^1^ In this case report, we describe two sisters with severe scoliosis, both carrying novel compound heterozygous variants in the *TTN* gene, yet presenting distinct clinical phenotypes, adding to the growing body of evidence linking *TTN* mutations to scoliosis and other titin-related disorders.

The older sister (patient 1) developed scoliosis at age 4 that progressed rapidly despite conservative treatment (Fig. 1A, B). She received spine fusion surgery in 2013 at age 11. She had no breathing problems, and preoperative echocardiography showed mild mitral valve prolapse with no sign of cardiomyopathy. However, she experienced sudden cardiac arrest intraoperatively but was successfully resuscitated. The younger sister (patient 2) also developed scoliosis at age 4 with rapid progression and was presented for spine surgery at age 12 in 2021 (Fig. 1C, D). Different from patient 1, patient 2 had progressively worsening exertional dyspnea with significantly poorer activity tolerance than peers. Preoperative evaluation revealed severely compromised pulmonary function as 19 % of predicted forced vital capacity (0.37 L), and arterial blood gas revealed type II respiratory failure (pH = 7.32, pCO_2_ = 70 mmHg, pO_2_ = 56 mmHg). She had a normal cardiac assessment otherwise. She underwent spinal fusion surgery successfully after thorough preoperative optimization. Spine magnetic resonance imaging showed asymmetric paraspinal muscle atrophy with fatty infiltration in both patients (Fig. 1E, F). Paraspinal muscle biopsy of patient 2 showed chronic myopathy (Fig. 1G–J). Both patients recovered well at follow-up, though patient 2 still required intermittent noninvasive ventilation support despite improved activity tolerance.

**Figure 1:**
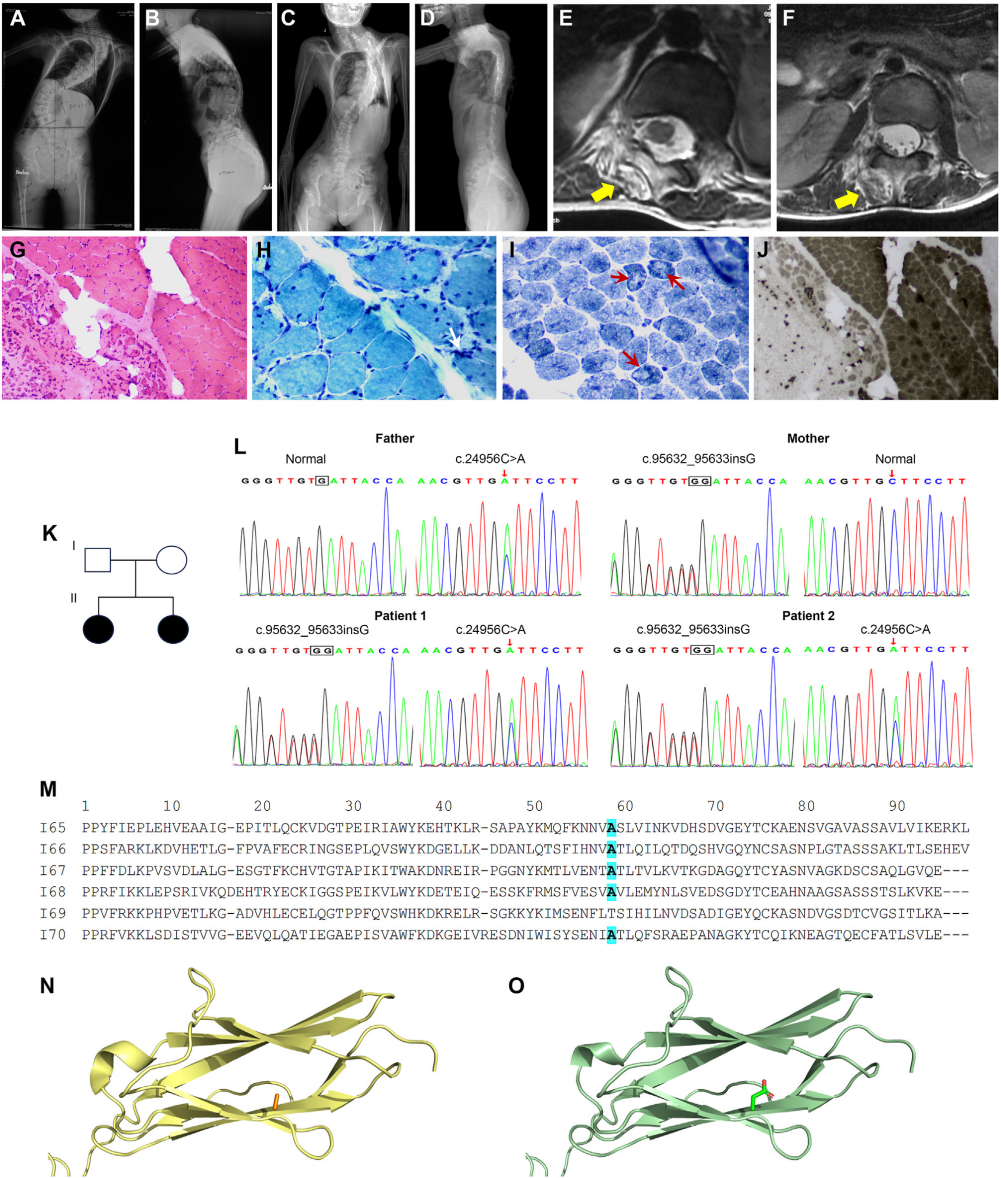
Clinical, pathological, and molecular features of the patients. **(A**–**D)** Preoperative X-rays showed severe scoliosis in patient 1 (A, B) and patient 2 (C, D). **(E, F)** Lumbar spine magnetic resonance imaging indicated asymmetric paraspinal muscle atrophy with fatty infiltration (yellow arrows) in patient 1 (E) and patient 2 (F). **(G**–**J)** Paraspinal muscle histopathology of patient 2 revealed increased muscle fiber size variation with fiber degeneration and necrosis in hematoxylin and eosin staining (G); subsarcolemmal cytoplasmic bodies (white arrow) in modified Gomori trichrome staining (H); “moth-eaten” fibers with reduced oxidative reaction in central areas (red arrows) in nicotinamide adenine dinucleotide–tetrazolium reductase staining (I); and unevenly distributed muscle fiber types in ATPase staining (Ph = 4.35): type I fibers was predominant in areas with mild atrophy, mixed with very few type II fibers; while type II muscle fibers predominance was found in severe atrophy areas (J). **(K)** Family pedigree. Filled symbols are affected and open symbols indicate unaffected members. **(L)** Sanger sequencing confirmed the two novel variants in *TTN* (c.95857_95858insG and c.25181C > A). **(M)** The missense variant (c.25181C > A, p.A8319D) is located in a highly conservative site in the I65 motif of titin. **(N, O)** Homology models show a small and non-charged alanine residue in wild-type titin (N), but a relatively large and negatively charged aspartate residue in A8319D (O).

Considering two severe scoliosis patients with different comorbidities occurred in the same family, a genetic examination was referred. Whole-exome sequencing was performed on the two affected sisters and their asymptomatic parents (Fig. 1K). Bioinformatic analysis revealed two potentially harmful novel bi-parentally inherited compound heterozygous *TTN* variants in both sisters. According to American Council of Medical Genetics (ACMG) guidelines, the maternally inherited truncating frameshift *TTN* variant, NM_001267550.2:c.95632_95633insG (p.D31878Gfs∗14) in exon 344 was classified as pathogenic, and the paternally inherited missense *TTN* variant, NM_001267550.2:c.24956C > A (p.A8319D) in exon 86 was classified as likely pathogenic. Both variants were absent from the gnomAD (https://gnomad.broadinstitute.org/) and 1000 genomes (https://www.internationalgenome.org) databases at the time of this study and were validated by Sanger sequencing (Fig. 1L).

Pathogenic *TTN* variants are associated with a wide spectrum of neuromuscular disorders with high phenotypic and genetic heterogeneity. Mutations in exon 344 encoding the myosin-binding fibronectin-3 (FN3) domain 119 in the A-band region of the *TTN* gene have been widely reported to be related to hereditary myopathy with early respiratory failure (HMERF), one of the major types of titinopathy.^1^ HMERF, usually characterized by adult-onset respiratory failure with proximal and/or distal muscle weakness, is mainly reported in association with missense mutations in exon 344 of *TTN*.^1^ However, the novel compound heterozygous *TTN* gene variants we identified in this study include a truncating frameshift *TTN* variant in exon 344, which could lead to a loss of function mutation of titin (D31878Gfs∗14) and be potentially linked with a more deleterious phenotype. In this study, the clinical manifestations, imaging results, and histopathological findings of patient 2 were generally compatible with HMERF, but she presented with significantly earlier onset respiratory insufficiency and much more severe scoliosis compared with previously reported HMERF cases. In addition, titin-truncating variants have been reported with dilated cardiomyopathy.^1^ Patient 1 experienced cardiac arrest during spine surgery despite normal cardiac evaluation, and the possibility of undiscovered cardiomyopathy cannot be ruled out though without direct evidence.

The second titin mutation identified in the sisters, the paternally inherited missense *TTN* variant in exon 86 (A8319D), is classified as likely pathogenic according to ACMG guidelines. However, mutations in exon 86 of *TTN* are far less reported than exon 344 variants and the significance remains unclear. We generated a homology model of the human point mutant titin based on the wild-type structure (PDB ID: 3B43) by using SwissModel.^2^ To further analyze how the identified variants impair the protein's function, the structure was visually inspected, and regions of interest were labeled using PyMOL (Schrödinger, 2015). The A8319D model was constructed based on the solved X-ray crystallographic structure as previously determined.^3^ The missense variant A8319D identified in individuals from our study is located at the b-strand E in the I65 motif of titin, which is a component of the poly-Ig tandems from the spring region of titin that contributes to the sarcomere elasticity.^3^ Structure-based sequence alignment reveals the high conservation of this site among the 6 Ig domains of I65–I70 (Fig. 1M). The side chain of A8319 points to the inner space of the I65 motif, which is a hydrophobic core (Fig. 1N). The wild-type alanine residue is small and non-charged while replacing it with the relatively large and negatively charged aspartate residue could potentially disturb the stability of the surrounding amino acid residues (Fig. 1O). Because of steric hindrance and changes in charge properties, A8319D may exert an abnormal impact on the function of titin.

Both novel *TTN* gene variants found in this study are suspected to affect titin function. Carrying these compound heterozygous *TTN* gene variants could be associated with severe skeletal muscle diseases, as observed in the two sisters. Despite sharing the same *TTN* variants and both having severe juvenile-onset scoliosis, their clinical presentations differed: patient 1 experienced intraoperative cardiac arrest, while patient 2 developed HMERF at a much younger age, highlighting the complex etiology of scoliosis and the accompanied myopathies. These differing phenotypes suggest that additional genetic or environmental factors may contribute to the observed differences. Whole-exome sequencing revealed other potentially deleterious variants in genes including *LAMA2*, *EPG5*, *FBN1*, *BRF1*, *ACADSB*, and *EPHA10*, each potentially affecting distinct metabolic and functional pathways. Both sisters share mutations in *TTN*, *EPG5*, *LAMA2*, *FBN1*, and *BRF1*, while *ACADSB* and *EPHA10* variants were uniquely detected in patient 2 (Table S1). Notably, mutations in *LAMA2* and *FBN1* may introduce additional complexities to the sisters' clinical presentation. *LAMA2* mutations are also known to be associated with muscular dystrophy, while *FBN1* mutations are linked to connective tissue abnormalities, including Marfan syndrome, which may also lead to scoliosis. Previous studies have reported cases with concurrent *TTN* and *LAMA2* mutations, highlighting overlapping features of muscular dystrophy.^4^^,^^5^ Though the compound heterozygous missense mutations in *LAMA2* and the heterozygous missense mutation in *FBN1* identified in this study are classified as variants of uncertain significance as per ACMG guidelines and have not been reported in patients with classic *LAMA2*-related muscular dystrophy or Marfan syndrome, their presence alongside *TTN* mutations may exacerbate the clinical manifestations observed in our patients. While the *TTN* mutations likely play a primary role, these additional mutations may contribute independently or synergistically to the phenotypic differences, suggesting a multifactorial basis.

Nevertheless, due to the small pedigree in this study and the lack of functional testing for each variant, the exact role of these mutations remains hypothetical. Further studies with larger cohorts and functional experiments are needed to validate and expand upon our findings, particularly regarding the genotype–phenotype correlation mechanisms.

## Ethics declaration

The study was approved by the Ethics Committee of Peking Union Medical College Hospital (I-24PJ0094) and written consent for publication was obtained.

## Author contributions

**Huaiyuan Wang:** Writing – original draft. **Shengjie Li:** Writing – original draft, Methodology, Investigation. **Weiyun Chen:** Writing – review & editing, Funding acquisition, Conceptualization. **Jianxiong Shen:** Writing – review & editing, Conceptualization.

## Funding

This study was supported by the 10.13039/501100001809National Natural Science Foundation of China (No. 82272446).

## Conflict of interests

The authors have no conflict of interests to declare.
