# Supplementary material for: Novel compound heterozygous TTN gene variants with additional potential contributory mutations in two sisters with severe scoliosis: A case report
Source: Genes Dis. 2024 Dec 3;12(3):101477. doi: 10.1016/j.gendis.2024.101477 (PMC11804530; doi:10.1016/j.gendis.2024.101477)
Supplement: Multimedia component 1 [file mmc1.docx]

**Materials and Methods**

**Clinical examination**

The clinical data were obtained from outpatient clinic visits and inpatient medical records. Demographics, perioperative laboratory test results, and intraoperative data were collected using our institutional database. For preoperative evaluation, imaging studies including X-rays, CT (with 3D reconstruction) and MRI of spine were performed. Blood tests including complete blood count, liver and renal function tests, and coagulation studies were completed. Arterial blood gas and pulmonary function test were applied for respiratory evaluation. Electrocardiogram and echocardiography were performed for cardiovascular assessment. Skeletal muscle function was assessed by electromyography and nerves conduction velocity. The study was approved by the Ethics Committee of Peking Union Medical College Hospital (I-24PJ0094).

**Pathology study**

Paraspinal muscle specimen of Patient 2 obtained during her spine surgery underwent histopathology examination with informed consent. Hematoxylin and eosin staining was routinely performed. Modified Gomori trichrome staining was performed for the diagnosis of muscular disorders. Nicotinamide adenine dinucleotide–tetrazolium reductase staining was used to assess the metabolic activity and distribution of oxidative enzymes in muscle fibers. ATPase staining was performed to differentiate muscle fiber types based on their ATPase activity.

**Genetic analysis**

1. Sample collection: Blood samples from both patients and their parents were collected after obtaining informed consent and were treated with EDTA.

2. DNA extraction: Genomic DNA was extracted using the Blood Genome Column Medium Extraction Kit (Kangweishiji, China) according to the manufacturer’s instructions. The quality of the extracted DNA was assessed using the Qubit 2.0 fluorometer and 0.8% agarose gel electrophoresis for further analysis.

3. Whole exome library construction: Exome enrichment targeting protein-coding regions was performed using the xGen Exome Research Panel v2.0 (IDT, Iowa, USA). This panel includes 429,826 individually synthesized and quality-controlled probes, covering 39 Mb of protein-coding regions (19,396 genes) of the human genome, with a total tiled probe space of 51 Mb.

4. Sequencing: High-throughput sequencing was carried out using MGI DNBSEQ-T7 sequencing instruments (paired-end sequencing with a read length of 150 bp). At least 99% of the target sequences were successfully sequenced. The sequencing process was performed by Beijing Chigene Translational Medicine Research Center Co., Ltd, 100875, Beijing. The sequencing data is summarized in Supplementary Table 2.

5. Bioinformatics analysis:

- **Quality Control:** Raw data were processed by fastp for adapters removing and low-quality reads filtering.
- **Variants calling:** T Paired-end reads were aligned to the Ensemble GRCh37/hg19 reference genome using the Burrows-Wheeler Aligner (BWA). Base quality score recalibration, as well as SNP and short indel calling, were performed using GATK. Based on sequence depth and variant quality, high-confidence SNPs and indels were identified, and reliable variants were obtained.
- **Variants annotation and pathogenicity prediction**: Variants were annotated using an online system developed by Chigene (www.chigene.org), which provides minor allele frequencies (MAFs) from various databases and predicts the pathogenicity of each gene variant based on ACMG guidelines. The system incorporates software for conservation analysis and protein structure prediction. Databases used for MAF annotation include 1,000 Genomes, dbSNP, ESP, ExAC, and Chigene’s in-house database. Predictive software tools such as Provean, SIFT, PolyPhen2_hdiv, PolyPhen2_hvar, MutationTaster, M-Cap, and REVEL were used for protein structure variation prediction. MaxEntScan, dbscSNV, and GTAG were employed for predicting the functional effects of variants on splicing sites. Pathogenicity annotations prioritized according to ACMG guidelines were confirmed with OMIM, HGMD, and ClinVar databases.

**Protein structure study**

The homology model of the human point mutant titin was generated by using SwissModel based on the wild-type structure (PDB: 3B43)^4^. The A8319D model was constructed based on the solved X-ray crystallographic structure as previously determined^5^. The structure was further visually inspected using PyMOL (Schrodinger, 2015) and regions of interest were labeled.

**Supplementary Table 1.** All the potentially deleterious mutations identified in the two patients

| **Gene** | **Nucleotide** | **Amino acid** | **ACMG classification** | **Patient 1** | **Patient 2** | **OMIM** | **Possible phenotype** |
| --- | --- | --- | --- | --- | --- | --- | --- |
| *TTN* | c.95632_96533insG | p.D31878Gfs*14 | Pathogenic | heterozygous | heterozygous | 604145  608807  603689  600334 | Muscular dystrophy  Cardiomyopathy  Respiratory failure |
| *TTN* | c.24956C>A | p.A8319D | Likely pathogenic | heterozygous | heterozygous |  |  |
| *LAMA2* | c.3616A>G | p.T1206A | Variants of uncertain significance | heterozygous | heterozygous | 607855  618138 | Muscular dystrophy |
| *LAMA2* | c.4993G>A | p.G1665R | Variants of uncertain significance | heterozygous | heterozygous |  |  |
| *EPG5* | c.2683A>G | p.I895V | Variants of uncertain significance | heterozygous | heterozygous | 242840 | Vici syndrome |
| *FBN1* | c.4894C>T | p.R1632C | Variants of uncertain significance | heterozygous | heterozygous | 614185  154700 | Geleophysic dysplasia  Marfan syndrome |
| *BRF1* | c.439+1G>A | N/A | Likely pathogenic | heterozygous | heterozygous | 616202 | Cerebellofaciodental syndrome |
| *ACADSB* | c.275C>G | p.S92*,341 | Likely pathogenic | wildtype | heterozygous | 610006 | 2-methylbutyrylglycinuria |
| *EPHA10* | c.2123G>A | p.R708Q | Likely pathogenic | wildtype | heterozygous | 620283 | Deafness |

**Supplementary Table 2.** Genomic sequencing data summary

|  | **Patient 1** | **Patient 2** | **Father** | **Mother** |
| --- | --- | --- | --- | --- |
| Raw data output (Mb) | 7818.24 | 6885.38 | 10892.29 | 15161.34 |
| Target region length (bp) | 34M | 34M | 34M | 34M |
| Probe coverage length (bp) | 42.5M | 42.5M | 42.5M | 42.5M |
| Exome coverage (%) | 99.50 | 99.50 | 99.70 | 99.70 |
| Average depth >10X coverage (%) | 98.30 | 98.30 | 98.70 | 99.00 |
| Average depth >20X coverage (%) | 96.70 | 96.60 | 97.70 | 98.50 |
| Average depth >30X coverage (%) | 94.20 | 93.60 | 96.40 | 97.90 |
| Exome coverage of *TTN* (%) | 100.00 | 100.00 | 100.00 | 100.00 |
